# Verification and application of deep learning models in daily sports activities of teenagers

**DOI:** 10.1371/journal.pone.0322166

**Published:** 2025-06-04

**Authors:** Lei Shi

**Affiliations:** Sports Department, Jiangsu College of Engineering and Technology, Nantong, Jiangsu, China; Ningbo University, CHINA

## Abstract

With the development of smart wearable devices and deep learning (DL) technology, the monitoring and analysis of daily sports activities of teenagers face new opportunities. At present, traditional CNN (Convolutional Neural Network) models are mostly used for recognition in daily sports activities. It is difficult to capture the temporal relationship between action sequences, and the ability to express important features is weak, resulting in poor recognition accuracy. This paper took badminton as the object, based on the VGG16 (Visual Geometry Group 16) model, and adopted the advantages of the bidirectional learning time series information of the BiLSTM (Bidirectional Long Short-Term Memory) model and the channel and regional feature representation of the CBAM (Convolutional Block Attention Module) module to verify and apply the recognition of badminton movements in daily sports for teenagers. The study first built and optimized the baseline model VGG16, removed the last three fully connected layers, and used VGG16 to extract the deep features of each frame of video image and output feature maps. The CBAM module was then embedded after the last convolutional layer of the VGG16 network, and the feature maps optimized by CBAM were flattened into a time series input vector. Finally, the BiLSTM model is introduced, and the CBAM and BiLSTM are connected in a cascade manner to capture the information of the previous and next dependencies in the video frame sequence and output the action classification results of badminton. The experiment is based on the badminton training dataset in the public dataset Roboflow to explore the action recognition performance in badminton in daily sports activities of teenagers. Experimental results show that the recognition accuracy of the VGG16-BiLSTM-CBAM model has reached 0.98, which is 0.08 higher than the benchmark model VGG16, and F1 has reached 0.96. Experimental results show that combined with the DL model VGG19 and the sequential model BiLSTM, the attention CBAM module can significantly improve the performance of action recognition in youth badminton, promote the safe conduct of sports activities, and provide a good reference for incorrect postures.

## 1. Introduction

As the country pays more attention to the physical health of young people, the monitoring and analysis of young people’s daily physical activities has gradually become an important means to improve their health level and the effectiveness of physical education [1–2]. At present, some CNN-based models have achieved certain results in motion recognition, but they often ignore the temporal relationship between actions, and the detailed feature representation in motion actions is not accurate enough. The accuracy and application effect of motion recognition have been significantly improved. How to use DL models, especially temporal information processing and feature enhancement technologies, to improve the accuracy of identifying teenagers’ daily sports activities has become an urgent problem to be solved.

In the past few years, with the rapid development of science and technology, deep learning has made significant progress in many fields, especially in computer vision and motion recognition. Studies have shown that deep learning methods have achieved certain results in gait pattern recognition and biomechanical analysis. Xu D et al. adopted a new method that can effectively realize the recognition of human gait patterns and provide inspiration for sports and clinical gait analysis [3]. They also studied how to accurately predict ACL force and improve the effectiveness of prediction by biomechanical landing patterns before and after fatigue [4]. The recognition and analysis of youth sports activities not only involves health monitoring, but also directly affects the reform of physical education and the implementation of personalized training. In the field of badminton, the model is currently weak in expressing important features, and accurately capturing the details in the movement is of great significance to improving the effect of youth sports monitoring and providing guidelines for the healthy life of young people.

In order to improve the accuracy of action recognition in daily sports activities of teenagers, the experiment takes badminton as an example. Aiming at the shortcomings of traditional CNN models in capturing temporal relationships and feature expression, an improved method integrating DL and temporal modeling is adopted. The study extracts video frame features based on the VGG16 model, and enhances channel and regional feature representation by embedding the CBAM module. Finally, BiLSTM is combined to capture temporal dependency information and realize action classification. Experimental verification on public data sets shows that the VGG16-BiLSTM-CBAM (Visual Geometry Group 16-Bidirectional Long Short-Term Memory-Convolutional Block Attention Module) model significantly improves the accuracy and robustness of action recognition. This provides technical support for scientific analysis of youth sports activities and intelligent correction of wrong actions.

This study innovatively combines the VGG16 model with BiLSTM temporal modeling, and embeds the CBAM module to enhance feature expression, which solves the shortcomings of the traditional CNN model in capturing motion timing relationships and detailed features, and significantly improves the accuracy of youth badminton motion recognition.Paper’s contribution:

(1) In the field of badminton motion recognition, this study innovatively combines the VGG16 model with the BiLSTM time series model, and enhances the expressiveness of features by embedding the CBAM module. The application of the fusion model opens up a new stage in the field of badminton motion recognition. The research overcomes the limitations of traditional CNN models in processing time series data, especially in capturing the dependencies between action sequences, making action recognition in youth badminton more accurate.
(2) This article studies embedding the CBAM module in the VGG16 model to enhance the expression of channel and spatial features. Using this method, the model can focus more on key motion features in moving images, significantly improving the accuracy of motion action recognition.
(3) The experiment uses the badminton training dataset in the public dataset Roboflow to verify the effectiveness and practical application value of the proposed VGG16-BiLSTM-CBAM method, demonstrating the strong application potential of the method in daily sports activities of young people and promoting the progress of motion analysis and wrong posture recognition technology.

## 2. Related work

As the pillars of the country, young people have gradually become the focus of training. Many scholars have begun to identify the movements of young people in sports activities and have achieved a lot of research results. Sarwar M A, Lin K C and other scholars used a skeleton-based key frame detection framework to recognize the postures of badminton players based on OpenPose, greatly improving the accuracy of action recognition [5,6]. Liu L and other scholars used visual search technology to identify the third arm stroke of badminton players. The recognition response time was only 0.8s, which improved the recognition accuracy to a certain extent [7]. Ooi J H and other scholars combined wireless inertial sensors and neural networks to identify the batting posture of badminton players, and the recognition accuracy reached 97.69% [8]. Traditional CNN models are widely used in volleyball [9,10], tennis [11,12], and table tennis [13,14] in daily sports activities of teenagers, which significantly improves the performance of teenagers’ action accuracy recognition. The above-mentioned scholars used traditional CNN models, OpenPose, etc., to conduct research on adolescents’ daily movements and achieved certain results. Its experimental model has problems such as incomplete capture of temporal features and insufficient feature expression, making it difficult to cope with the diversity and complexity of daily sports of teenagers.

In recent years, in order to improve the accuracy of motion recognition, many scholars have begun to integrate different DL technologies, such as LSTM (Long Short-Term Memory), deep CNN, and attention mechanism. The VGG16 model is widely used in adolescent gait and action recognition. Compared with traditional shallow neural network models, it captures deeper features and improves the model’s recognition accuracy of adolescent sports movements [15–17]. Li X combined LSTM and attention mechanism for posture recognition in volleyball sports, significantly improving the ability to capture the temporal sequence of action sequences [18]. Ruiye Z, Hassan N and other scholars applied the BiLSTM model to action classification in videos such as volleyball sports, bidirectionally capturing the temporal relationship of video frames, and the recognition accuracy rate reached more than 96% [19,20]. Zhi J et al. introduced the CBAM module to improve the TSN network (Time-Sensitive Networking) and used it for badminton video action recognition, with an average accuracy of 91.6% [21]. Peng X et al. combined CBAM and C3D (Convolutional 3D) for posture recognition in swimming, with an accuracy improvement of 14.50% compared to the traditional C3D model [22]. The above scholars used VGG16 model, BiLSTM model, CBAM and other attention modules to identify daily sports activities of teenagers, and achieved certain results, but failed to effectively integrate and apply them in sports action recognition, and they were rarely used in the recognition of badminton sports in daily sports activities of teenagers, leaving a lot of research gaps.

## 3. Design of sports activity recognition model

### 3.1. VGG16 Model Design

VGG16 is a classic CNN architecture, which contains 13 convolutional layers and 3 fully connected layers. VGG16 uses the gradual extraction of spatial features from low-level to high-level, has strong image processing capabilities, and is particularly suitable for image classification and recognition tasks [23–25]. In this paper, in order to adapt to the action recognition task of youth badminton sports, the fully connected layer of VGG16 is removed to reduce the number of model parameters.

The fully connected layer contains a large number of trainable parameters, which can easily make the model too complex when processing high-dimensional features, affecting the training effect. The study replaced the fully connected layer with a global average pooling layer, so that the model can retain the spatial information extracted by the convolution layer, while reducing the number of parameters and improving the generalization ability of the model. The improvement here in this paper makes the model more lightweight and meets the needs of the youth badminton action recognition task. It also effectively avoids the overfitting problem of high-dimensional features, which helps to improve the training efficiency and performance of the model.

In VGG16, the calculation formula of the convolution operation of each layer is shown in formula (1).


$$\begin{array}{c} y=g(W*z+a) \end{array}$$ (1)

z represents the input image or the output feature map of the previous layer, and W represents the convolution kernel. * represents the convolution operation, and a represents the bias term.

During the processing, VGG16 uses the maximum pooling layer for downsampling and increases the nonlinear ability through the ReLU activation function, so that the model can capture more complex features. The ReLU activation function is expressed as formula (2).


$$\begin{array}{c} g(z)=max(0,z) \end{array}$$ (2)

g(z) represents the ReLU activation function.

For the VGG16 model, a maximum pooling layer is used after each convolutional layer to reduce the size of the feature map. The calculation formula of the pooling operation is shown in formula (3).


$$\begin{array}{c} y=max(z_{1,1}{,...z}_{i,j}) \end{array}$$ (3)

$z_{i,j}$ represents the pixel value of the pooling window.

In order to solve the fitting risk of the VGG16 model in badminton sports action recognition, this paper introduces a global average pooling layer to replace the fully connected layer after the last convolution output feature map of the VGG16 model [26]. The calculation formula of the global average pooling layer is shown in formula (4).


$$y_{c}=\frac{1}{H\times W}\sum_{i=1}^{H}\sum_{j=1}^{W}z_{i,j,c}$$ (4)

$y_{c}$ represents the mean of the cth channel, and $z_{i,j,c}$ represents the feature value of the corresponding position in the feature map.

In the recognition of badminton movements, features of different scales include different feature information. The overall posture of youth sports requires a wider field of view, while detailed movements, such as the moment of hitting the ball, rely on fine local features. This paper adopts a multi-scale fusion strategy [27–29], outputs multiple feature maps in the middle layer of the VGG16 model, and uses convolution operations to fuse all feature maps to output feature information of different scales.

In this paper, the specific implementation of the multi-scale feature fusion strategy is to output multiple feature maps of different scales in the middle layer of the VGG16 model and fuse these feature maps through convolution operations. The VGG16 model obtains feature information of different levels and scales after multiple convolution layers, where the information covers the overall motion posture and local detail features. By fusing features of different scales, this paper’s model can more comprehensively capture the details and global information in the motion when processing badminton motion recognition, thereby improving the accuracy and robustness of recognition.

After being processed by VGG16, the output feature map is a three-dimensional tensor H*W*C. In order to facilitate the processing of the BiLSTM model, the study flattens its features into a vector so that each dimension of the feature becomes a time step of a time series, as shown in formula (5).


$$\begin{array}{c} z_{t}=Re(H,W,C)\to R^{(H*W)*C} \end{array}$$ (5)

$z_{t}$ represents the flattened vector expression.

### 3.2. Construction of BiLSTM model

BiLSTM is a DL model that can simultaneously learn temporal dependencies from the forward and backward directions of a sequence. It is mainly used to process and predict contextual information in sequence data [30,31]. This paper introduces the BiLSTM model to capture the temporal information in youth badminton sports and learn the contextual information during the sports process.

The BiLSTM model consists of two LSTM units, one is the forward LSTM, which runs from the beginning to the end of the sequence; the other is the reverse LSTM, which runs from the end to the beginning of the sequence [32]. Each LSTM unit includes three gates: input gate, forget gate, and output gate. In the forget gate, the calculation is shown in formula (6).


$$b_{t}=\sigma (W_{b}\cdot [h_{t-1},z_{t}]+\gamma_{b}$$ (6)

$z_{t}$ represents the input vector, $W_{b}$ represents the weight matrix of the forget gate, $\gamma_{b}$ represents the bias vector, and σ represents the Sigmoid activation function.

The calculation formula of the input gate is shown in formula (7).


$$j_{t}=\sigma (W_{j}\cdot [h_{t-1},z_{t}]+\gamma_{j}$$ (7)

$W_{j}$ represents the weight matrix of the input gate, and $\gamma_{j}$ represents the bias vector.

The calculation formula of the candidate state is shown in formula (8).


$${\hat{D}}_{t}=tanh(W_{D}\cdot [h_{t-1},z_{t}]+\gamma_{D}$$ (8)

tanh represents the hyperbolic tangent activation function.

The calculation formula of the output gate is shown in formula (9).


$$p_{t}=\sigma (W_{p}\cdot [h_{t-1},z_{t}]+\gamma_{p}$$ (9)

The calculation formula of cell state is shown in formula (10).


$$D_{t}=b_{t}\cdot D_{t-1}+j_{t}\cdot {\hat{D}}_{t}$$ (10)

The calculation formula of the hidden state is shown in formula (11).


$$h_{t}=p_{t}\cdot tanh(D_{t})$$ (11)

$h_{t}$ represents the hidden state.

In the BiLSTM model, for the forward LSTM, the sequence data is calculated step by step from 1 to T, while in the reverse LSTM, the sequence data is calculated from T to 1. The BiLSTM model performs a merge operation after the hidden states calculated by the forward and reverse LSTMs at each time step are merged. The bidirectional hidden state representation is shown in formula (12).


$${\hat{h}}_{t}=[h_{t}^{f},h_{t}^{b}]$$ (12)

Among them, $h_{t}^{f}$ represents the hidden state of the forward LSTM at time step t, and $h_{t}^{b}$ represents the hidden state of the reverse LSTM at time step t.

After the hidden state output by BiLSTM, a fully connected layer is connected to identify and classify the badminton sports movements of young people. The fully connected layer uses the softmax function for classification, and the calculation formula is shown in formula (13).


$$\hat{p}=softmax(W_{bD}\cdot {\hat{h}}_{T}+\gamma_{bD})$$ (13)

${\hat{h}}_{T}$ represents the BiLSTM output of the last time step, $W_{bD}$ represents the weight of the fully connected layer, and $\gamma_{bD}$ represents the bias of the fully connected layer. $\hat{p}$ represents the probability of the badminton action category.

### 3.3. CBAM module

The CBAM module is a lightweight attention mechanism that includes channel attention and spatial attention. It improves the model’s ability to focus on key features by gradually optimizing the channel dimension and spatial dimension of the feature [33,34]. In the channel attention part, CBAM uses global maximum pooling and average pooling to extract channel global information, and uses a shared fully connected layer to generate channel weights. In the spatial attention part, CBAM uses pooling operations to compress channel information and combines convolutional networks to generate spatial weights.

#### 3.3.1. Channel attention mechanism design.

The channel attention mechanism uses the channel importance of different feature maps to assign weights to improve the network’s perception of key channels. In the channel attention mechanism, it uses global average pooling (GAP) and global maximum pooling (GMP) to reduce the dimension of the input feature map G and output vectors $G_{a}^{c}$ and $G_{m}^{c}$ that describe the global information of the channel. The calculation formulas of $G_{a}^{c}$ and $G_{m}^{c}$ are shown in formulas (14) and (15) respectively.


$$G_{a}^{c}=\frac{1}{H\cdot W}\sum_{i=1}^{H}\sum_{j=1}^{W}G(i,j,c)$$ (14)

$$G_{m}^{c}{=}_{i,j}^{\max}G(i,j,c)$$ (15)

c is between 1 and C, $G_{a}^{c}$ represents the result of global average pooling, and $G_{m}^{c}$ represents the result of global maximum pooling.

After the global pooling operation, the channel attention mechanism inputs $G_{a}^{c}$ and $G_{m}^{c}$ into the fully connected network with shared weights respectively, and gradually reduces the dimension through two layers. The calculation formula is shown in formula (16).


$$N_{c}(G)=\sigma (W_{2}\cdot ReLU(W_{1}\cdot G_{a}^{c}+\alpha_{1})+\alpha_{2})+\sigma (W_{2}\cdot ReLU(W_{1}\cdot G_{m}^{c}+\alpha_{1})+\alpha_{2})$$ (16)

$N_{c}(G)$ represents the channel attention weight. $W_{1}$ and $W_{2}$ both represent the fully connected layer weight matrix, and $\alpha_{1}$ and $\alpha_{2}$ represent the bias vector.

In this paper, $N_{c}(G)$ and G are multiplied point by point according to the channel, and the feature map after the channel attention weighting is output, as shown in (17).


$$G_{c}^{\prime }=G⊙N_{c}(G)$$ (17)

⊙ represents point-by-point multiplication, and $G_{c}^{\prime }$ represents the feature map after channel attention weighting.

The channel attention mechanism in the CBAM module enhances the model’s ability to focus on key features by assigning importance weights to different channels. The channel attention mechanism first extracts the global information of the input feature map through global average pooling and global maximum pooling, and generates channel-level description vectors. These description vectors are processed through a fully connected network with shared weights, and the channel weights are output. The final model multiplies these weights with the feature map point by point to highlight the features of important channels and suppress irrelevant channel information.

#### 3.3.2. Spatial attention mechanism design.

In order to highlight the spatial characteristics of specific areas when teenagers play badminton, the study adopts a spatial attention mechanism to enhance the sensitivity to the target action position. The spatial attention mechanism uses global maximum pooling and global average pooling to compress $G_{c}^{\prime }$ in the channel dimension and output feature maps $G_{a}^{s}$ and $G_{m}^{s}$. The calculation formulas of $G_{a}^{s}$ and $G_{m}^{s}$ are shown in formulas (18) and (19) respectively.


$$G_{a}^{s}(i,j)=\frac{1}{C}\sum_{c=1}^{C}G_{c}^{\prime }(i,j,c)$$ (18)

$$G_{m}^{s}(i,j){=}_{c}^{\max}G_{c}^{\prime }(i,j,c)$$ (19)

In the channel dimension, the spatial attention mechanism uses $G_{a}^{s}$ and $G_{m}^{s}$ for splicing and extracts spatial attention through a 7*7 convolution kernel. The calculation is shown in formula (20).


$$N_{s}(G)=\sigma ({Conv}_{7*7}(G_{s}))$$ (20)

$N_{s}(G)$ represents the spatial attention weight, and $G_{s}$ represents the concatenated feature map.

The feature map after spatial attention weighting is shown in formula (21).


$$G_{s}^{\prime }=G_{c}^{\prime }⊙N_{s}(G)$$ (21)

$G_{s}^{\prime }$ represents the feature map after spatial attention weighting.

#### 3.3.3. Embedding of CBAM module.

As a component that integrates channel attention and spatial attention, the CBAM module enhances the expressiveness of target features in the feature extraction stage while maintaining compatibility with the subsequent temporal modeling layer. In order to improve the ability to express important features in badminton sports for young people, this paper embeds the CBAM module after the last convolution layer of VGG16 to make the feature extraction of each frame more distinguishable. The experiment uses this embedding method, and the CBAM module can effectively enhance the feature expression ability and time series modeling performance of the VGG16-BiLSTM (Visual Geometry Group 16-Bidirectional Long Short-Term Memory) model, providing important support for the action recognition of daily sports activities for young people.

The use of the CBAM module in the experiment will introduce additional computational operations, such as global pooling, fully connected layers, and convolutional layers. However, it is designed as a lightweight module with relatively simple operations and moderate computational complexity, which will not significantly increase the computational complexity of the model. By optimizing the attention of the channel and spatial dimensions only in the feature extraction stage, CBAM can enhance the model’s focus on key features while maintaining low computational overhead. In order to balance computational cost and model performance, this paper reduces the complexity of the model by embedding the CBAM module into the last convolutional layer of VGG16, and combines global average pooling instead of the fully connected layer to effectively control the amount of computation, ensuring that the model has high computational efficiency while improving recognition accuracy.

### 3.4. Fusion of VGG16-BiLSTM and CBAM modules

In the recognition task of youth badminton sports movements, in order to simultaneously capture the temporal characteristics and spatial characteristics of the action sequence and improve the perception of key features, this paper fuses the VGG16, BiLSTM and CBAM modules to construct a highly targeted feature extraction and temporal modeling architecture.

**Fig 1:**
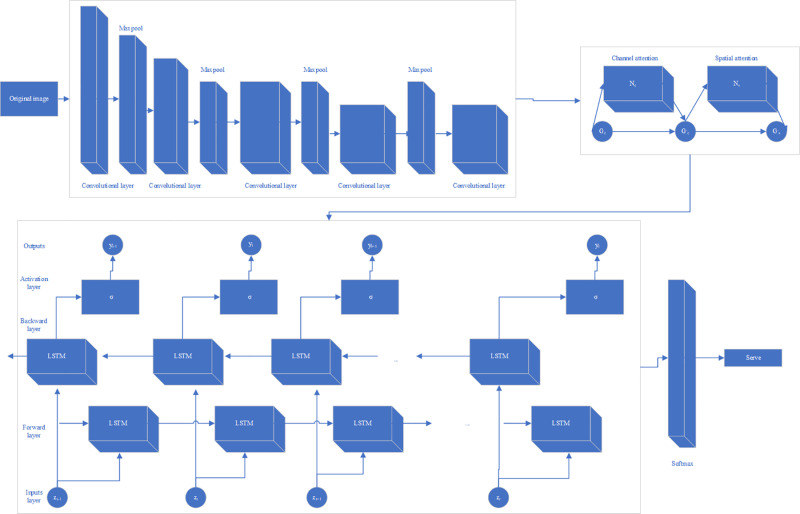
VGG16-BiLSTM-CBAM structure diagram.

The structure diagram of VGG16-BiLSTM-CBAM is shown in Fig 1.

In Fig 1, it can be seen that the study first embeds the CBAM module into the last convolutional layer of the VGG16 network to strengthen the model’s ability to focus on key areas and feature channels in badminton movements, and then flattens the feature map optimized by CBAM into a time series input vector and inputs it into the BiLSTM network time by time. CBAM and BiLSTM are fused in a cascade manner.

### 3.5. Model training and optimization

#### 3.5.1. Loss function design.

In the training and optimization of the model, this paper uses a weighted combination of the cross entropy loss function and the center loss to measure the loss. The calculation formula of the combined loss function is shown in formula (22).


$$\begin{array}{c} L=-\frac{1}{N}\sum {\mathrm{\backslash nolimits}}_{i=1}^{N}\sum {\mathrm{\backslash nolimits}}_{k=1}^{K}I[y_{i}=k]log{\hat{p}}_{k}+\frac{1}{2}\sum {\mathrm{\backslash nolimits}}_{i=1}^{N}{\left||h_{i}-c_{y_{i}}|\right|}^{2} \end{array}$$ (22)

I represents the indicator function, ${\hat{p}}_{k}$ represents the probability that the sample is predicted to be class k, and $c_{y_{i}}$ represents the center vector of class $y_{i}$.

The calculation formula of ${\hat{p}}_{k}$ is shown in formula (23).


$$\begin{array}{c} {\hat{p}}_{k}=\frac{exp(w_{k}^{T}h_{i})}{\sum_{j=1}^{K}exp(w_{j}^{T}h_{i})} \end{array}$$ (23)

$w_{k}^{T}$ represents the weight of category k, and $h_{i}$ represents the final output of BiLSTM.

In the center loss, $c_{y_{i}}$ is dynamically adjusted using momentum update, as shown in formula (24).


$$c_{k}\leftarrow c_{k}+\delta \sum_{i\in \{i|y_{i}=k\}}\left(h_{i}-c_{k}\right)$$ (24)

δ represents the learning rate.

#### 3.5.2. Model training.

During the training process, the experiment uses small batch stochastic gradient descent. The training steps are as follows:

(1) This paper first inputs each batch of samples into the model, and then processes them with VGG16, CBAM, and BiLSTM to output the final classification features.
(2) According to the predicted probability output by the model and the real label of the real badminton sports action, the cross entropy loss and center loss are calculated, and the gradient of the model parameters is calculated using automatic differentiation.
(3) The parameters are updated using the AdamW optimizer, and the calculation formula is shown in formula (25).

$$\theta_{t}=\theta_{t-1}-\frac{\zeta }{\sqrt{v_{t}}+\eta }\iota_{t}-\kappa \theta_{t-1}$$ (25)

η represents an extremely small parameter with a value of 10^−8^.

The hyperparameter settings are shown in Table 1.

**Table 1:** Hyperparameters.

Parameters	Value	Parameters	Value
Batch size	64	Hidden layer dimensions	512
Initial learning rate	0.001	Central loss weight	0.5
Weight decay coefficient	0.01	Dropout rate	0.5
CBAM channel attention scaling ratio	16	Optimizer	AdamW

In Table 1, the batch size is 64, the initial learning rate is 0.001, the weight decay coefficient is 0.01, and the CBAM channel attention scaling ratio is 16. The hidden layer dimension is 521, the center loss weight is 0.5, and the Dropout rate is 0.5.

#### 3.5.3. Model optimization.

To prevent the model from falling into the local optimum, the experiment uses the cosine annealing learning rate strategy [35–36] to dynamically adjust the learning rate during training, and sets an early stopping strategy. When there is no significant decrease in the data set for 10 consecutive epochs, the training is terminated early.

The calculation formula of the cosine annealing learning rate strategy is shown in formula (26).


$$\lambda_{t}=\lambda_{\min}+0.5(\lambda_{\max}-\lambda_{\min})(1+cos(\frac{t}{T}\pi ))$$ (26)

T represents the maximum number of iterations of training. $\lambda_{\max}$ is 0.001 and $\lambda_{\min}$ is 10^−6^.

In the cosine annealing learning rate strategy, a higher $\lambda_{\max}$ helps the model quickly explore the parameter space and accelerate convergence in the early stages of training, while a lower $\lambda_{\min}$ can avoid oscillation or instability caused by excessive learning rates in the later stages of training. If $\lambda_{\max}$ is too large or $\lambda_{\min}$ is too small, the model will converge too early in the early stages or be undertrained in the later stages. Reasonable $\lambda_{\max}$ and $\lambda_{\min}$ settings can effectively balance training speed and accuracy and promote robust convergence of the model.

In model training, the experiment adds Dropout to the hidden layer output of the BiLSTM module to reduce the risk of overfitting parameters, and introduces category weights in the loss function, which are dynamically adjusted according to the number of categories.

## 4. Action recognition experiment for youth badminton

### 4.1. Experimental data and preprocessing

The experimental data in this paper comes from the badminton training dataset in the public dataset Roboflow, which includes 20 videos in total. The actions include Serve, Smash shot, clear shot, Drive shot, and Drop shot. This paper randomly selected eight badminton training videos from the public dataset Roboflow, each of which is about 6 minutes long. The experiment uses the ten-fold crossover method to divide the dataset into a training set and a test set, and finally takes the average as the final result.

In the Roboflow dataset, it not only includes badminton training data for teenagers, but also covers data for other age groups. The dataset covers badminton actions such as serving, smashing, clearing, etc. in youth sports, including the main action characteristics of badminton, and the performance and rules of these actions are common to athletes of different ages. It is feasible to use the Roboflow dataset for youth badminton action recognition in this paper. Especially in the design of the model, by combining time series modeling and attention mechanism, the characteristics of young athletes when performing these actions can be better captured.

For the Roboflow dataset, from the perspective of practical application, the categories of the dataset cover the five common action types in badminton, which can provide diverse training data. In order to avoid the deviation caused by category imbalance, the experiment balances the data through data enhancement and other technologies. Regarding generalization ability, this paper adopts a ten-fold cross-validation strategy to effectively evaluate the stability and generalization performance of the model, ensure that the performance of the model in different environments and data distributions is not affected by overfitting, and improve the reliability of practical applications.

Preprocessing:

(1) Video segmentation and video frame extraction

The experiment uses the DTW (Dynamic Time Warping) algorithm [37] to segment the badminton actions in the video, convert the original video into a frame sequence, and automatically annotate the key action moments based on the motion trajectory features. After segmentation, this paper uses a fixed frame rate of 25 frames per second to extract video frames. In each action interval, only the continuous frames between the start and end key frames of the action are retained. For action segments with insufficient duration, the time interpolation algorithm is used to supplement the frames.

(2) Data enhancement

In order to increase the robustness of the model, the experiment performs data enhancement processing such as rotation and translation on the extracted frame sequence. The rotation processing is uniformly processed by counterclockwise rotation of 30 degrees, and the translation is uniformly processed by random selection.

In the data enhancement method, the translation operation uses a random selection of [50,100] to effectively increase the diversity of training data, enabling the model to learn more features under different perspectives and positions, and improve its ability to adapt to changes in actual scenes. Through enhancement processing, this paper can better generalize the model to different actions and environments, reducing the risk of overfitting.

(3) Data standardization

The study uses zero mean unit variance standardization to normalize the pixel values of each frame so that the range is between 0 and 1. The calculation formula for normalization processing is shown in formula (27).


$$\varrho^{\prime }(x,y)=\frac{\varrho (x,y)-\tau }{\varsigma }$$ (27)

τ represents the mean of the frame image pixels, and ς represents the standard deviation.

### 4.2. Experimental environment

Hardware environment: Intel Xeon W-2295, NVIDIA A100, 256GB DDR4 (Double Data Rate 4), 2TB NVMe SSD (Solid State Drive).

Software environment: Windows 10, Python 3.9, OpenCV 4.8, Jupyter Notebook.

### 4.3. Evaluation indicators

Accuracy:


$$\begin{array}{c} Acuracy=\frac{TP+TN}{TP+TN+FP+FN} \end{array}$$ (28)

TP means true positive, TN means true negative, FP means false positive, and FN means false negative.

Precision:


$$\begin{array}{c} Precision=\frac{TP}{TP+FP} \end{array}$$ (29)

Recall:


$$\begin{array}{c} Recall=\frac{TP}{TP+FN} \end{array}$$ (30)

F1:


$$\begin{array}{c} F1=2\cdot \frac{Precision\cdot Recall}{Precision+Recall} \end{array}$$ (31)

### 4.4. Experimental design

Experimental Objectives:

(1) This paper explores whether the VGG16 model integrating BiLSTM and CBAM modules can effectively improve the accuracy of badminton action recognition.
(2) This paper studies whether the CBAM module reduces the interference of irrelevant background information in feature representation, and verifies whether the BiLSTM module can effectively capture the temporal dependency in the action sequence.

Experimental steps:

(1) Based on the collected badminton training videos, the DTW algorithm is first used to segment the badminton movements in the video and automatically annotate the key action moments based on the motion trajectory features.
(2) The video frame images are enhanced by counterclockwise rotation and random cropping, and the pixel values of each frame are normalized using zero-mean unit variance normalization.
(3) After preprocessing, the study first built and optimized the baseline model VGG16, removed the last three fully connected layers, and used VGG16 to extract the deep features of each frame of the video image and output it as a feature map.
(4) The experiment embedded the CBAM module after the last convolutional layer of the VGG16 network, and combined the CBAM optimized feature map to flatten it into a time series input vector.
(5) This paper introduces the BiLSTM model and connects CBAM and BiLSTM in a cascaded manner to realize bidirectional processing of input data to capture the information of the previous and next dependencies in the video frame sequence and output the final badminton action classification results.
(6) This paper statistically analyzes the accuracy, precision and other performance results of badminton action recognition and classification in daily sports activities of young people.

This paper selects VGG16-ConvLSTM, ResNet, InceptionV3 and C3D as comparison methods based on their wide application and good performance in image processing and action recognition tasks. VGG16-ConvLSTM combines the classic CNN and time series modeling capabilities, which is suitable for capturing time series information in action recognition. ResNet and InceptionV3, as deep residual networks and efficient convolutional neural network architectures, have advantages in image feature extraction respectively. The C3D model performs well in video action recognition, capturing spatiotemporal features through three-dimensional convolution. The experiment selects these models as comparison methods to comprehensively evaluate the performance improvement of the VGG16-BiLSTM-CBAM model in badminton action recognition, ensuring the fairness of the comparison and the reliability of the results.

## 5. Verification results in daily sports activities of young people

### 5.1. Confusion matrix analysis

**Fig 2:**
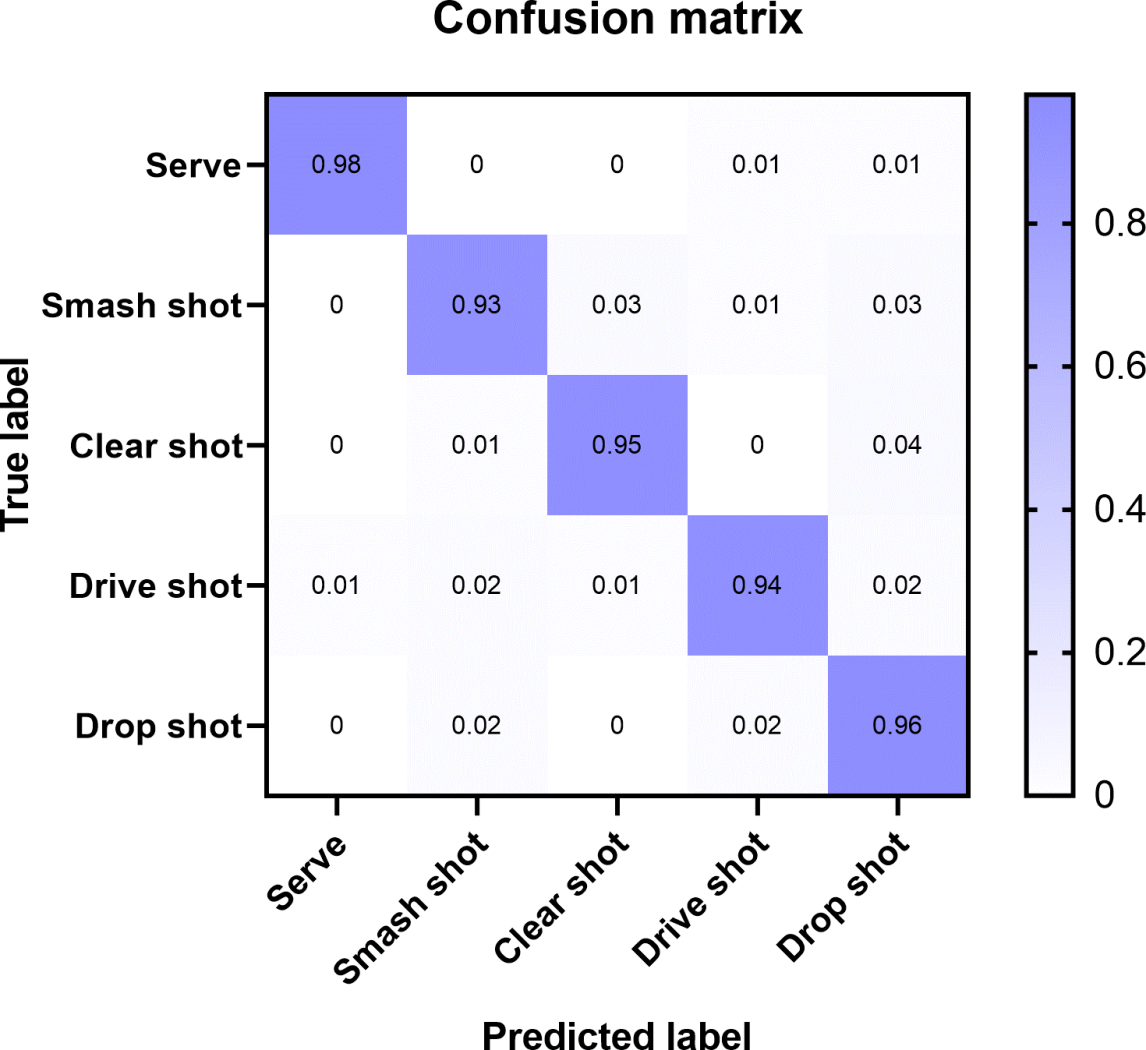
Confusion Matrix.

The confusion matrix results are shown in Fig 2.

In Fig 2, the horizontal axis represents the predicted labels, from left to right, Serve, Smash shot, Clear shot, Drive shot, Drop shot. The vertical axis represents the actual labels, from top to bottom, the same as the horizontal axis. Overall, all categories are between 0.93 and 0.98, among which the Serve action has the highest classification accuracy, reaching 0.98, and the Smash shot is only 0.93. In the Serve action, the predictions for Drive shot and Drop shot both reached 0.01. In the Smash shot, 0.03 was predicted as a clear shot, 0.01 was predicted as a Drive shot, and 0.03 was predicted as a Drop shot.

### 5.2. Badminton action recognition performance

**Fig 3:**
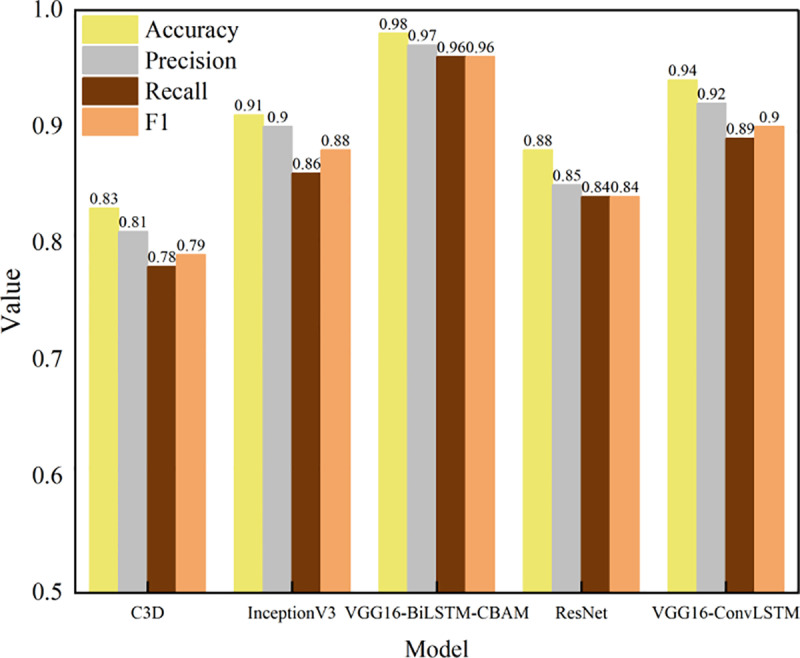
Badminton action recognition performance.

In order to further explore the recognition performance of badminton movements in daily sports activities of teenagers, the experimental model VGG16-BiLSTM-CBAM is compared with the comparison model in terms of accuracy, precision, recall rate, and F1 value. The results are shown in Fig 3. In Figure 3, the control models include VGG16-ConvLSTM (Visual Geometry Group 16-Convolutional Long Short-Term Memory), ResNet (Residual Network), InceptionV3, and C3D.

In Fig 3, from the accuracy and F1 value, VGG16-BiLSTM-CBAM performs best in badminton action recognition, reaching 0.98 and 0.96 respectively, which is 0.04 and 0.06 higher than VGG16-ConvLSTM. The ResNet model has an accuracy of 0.88 and an F1 value of 0.84, while the C3D model has the lowest accuracy of only 0.83 and an F1 value of only 0.79.

In terms of precision and recall, the precision and recall of VGG16-BiLSTM-CBAM are 0.97 and 0.96 respectively, which is significantly better than other control models. The precision of VGG16-ConvLSTM reaches 0.92 and the recall reaches 0.89. The precision of the InceptionV3 model reaches 0.9 and the recall reaches 0.86.

In summary, VGG16-BiLSTM-CBAM shows the best performance in the recognition of badminton movements. It has significant comprehensive advantages in feature extraction, time series modeling and attention mechanism. The BiLSTM module effectively captures the time series characteristics of badminton movements, while the CBAM attention mechanism enhances the focus on important spatial and channel features, significantly improving the overall recognition performance of the model.

### 5.3. Recognition accuracy in different noise and light intensity environments

**Fig 4:**
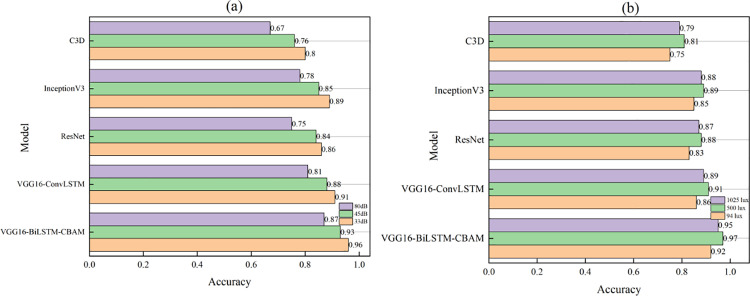
Recognition accuracy in different noise and light intensity environments.

Badminton is an important sport to improve the physical fitness of teenagers in their daily sports activities. This paper verifies the performance of the model in different noise and light intensity environments. The recognition performance under different noise and light intensity environments is shown in Fig 4. In Fig 4, different noises include low noise 33dB, medium noise 45dB, and high noise 80dB. Different light intensities include low light environment 94 lux, medium light intensity 500 lux, and high light intensity 1025 lux.

Fig 4(a) Recognition accuracy in different noise environments.

Fig 4(b) Recognition accuracy in different light intensity environments.

In Fig 4(a), for different noise environments, the impact of noise on the recognition performance of various models is relatively large. As the noise increases, the recognition accuracy decreases, and VGG16-BiLSTM-CBAM shows the best performance overall. In a low-noise 33dB environment, the accuracy of VGG16-BiLSTM-CBAM reaches 0.96, VGG16-ConvLSTM reaches 0.91, ResNet reaches 0.86, and C3D reaches 0.80. When the noise increases to medium noise (45dB) and high noise (80dB), the accuracy of the VGG16-BiLSTM-CBAM model drops to 0.93 and 0.87 respectively, but it is still better than other control models. The accuracy of VGG16-ConvLSTM is only 0.88 under medium noise and 0.81 under high noise.

In Fig 4(b), the recognition under different light intensity scenes is shown. VGG16-BiLSTM-CBAM achieves the highest accuracy of 0.97 in a medium light environment of 500 lux, which is 0.06 and 0.09 higher than VGG16-ConvLSTM and ResNet respectively. In low light environment of 94 lux and high light environment of 1025 lux, the accuracy of VGG16-BiLSTM-CBAM model remains at 0.92 and 0.95 respectively.

Overall, VGG16-BiLSTM-CBAM has good robustness and can adapt well to different noise and lighting environments. The CBAM module uses enhanced attention to key features to reduce the interference of noise and light changes on the recognition process. At the same time, BiLSTM’s modeling ability for time series enables it to maintain high recognition stability in complex lighting environments.

### 5.4. Comparison of model training time, response time, and parameter quantity

The training time, response time, and parameter amount comparison results of different models are shown in Table 2.

**Table 2:** Training time, response time, and parameter amount of different models.

Model	Training time (h)	Response time (ms)	Parameter quantity (M)
VGG16-BiLSTM-CBAM	2.5	45	35
VGG16-ConvLSTM	2.2	50	33
ResNet	1.5	42	25
InceptionV3	1.8	48	23
C3D	1.2	60	20

In Table 2, the training time of VGG16-BiLSTM-CBAM is 2.5 hours, which is the longest, while VGG16-ConvLSTM is only 2.2 hours and C3D is only 1.2 hours. BiLSTM is mainly used for time series modeling in this experiment, and its computational complexity is relatively high. In addition, the CBAM attention mechanism requires additional calculation of the weight distribution of the feature map, which increases the training time.

In terms of response time, VGG16-BiLSTM-CBAM performs better, only 45ms, achieving good, while C3D is as high as 60ms. It can be seen that the network architecture optimized in this paper has higher efficiency in the inference stage and is suitable for real-time motion action recognition applications. In terms of parameter quantity, VGG16-BiLSTM-CBAM has 35M parameters, VGG16-ConvLSTM has 33M parameters, and C3D has the least, only 20M. Overall, VGG16-BiLSTM-CBAM has a good balance in training time, response time, and parameter quantity among different models, but further lightweight processing of the model is needed.

### 5.5. Comparison of resource utilization of different models

**Fig 5:**
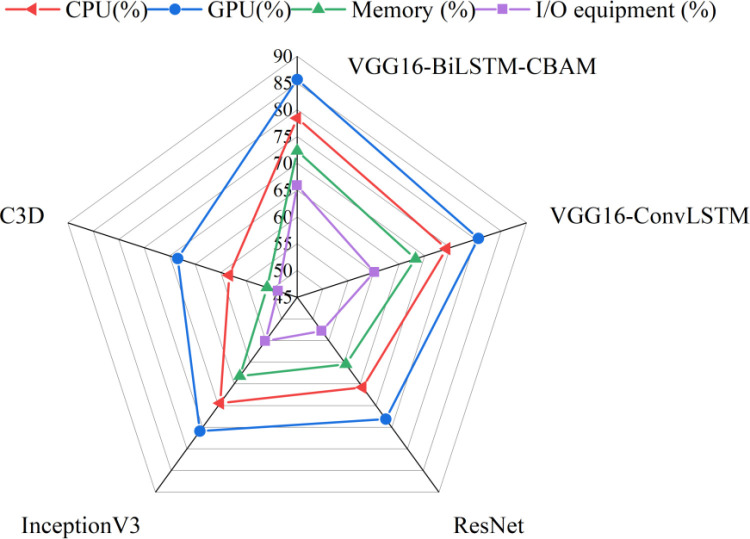
Resource utilization of different models.

In real-time sports of teenagers, good response time can enable them to adjust their sports posture in time and avoid injuries. The experiment statistically analyzed the resource utilization of the model from four aspects: CPU (Central Processing Unit), GPU (Graphics Processing Unit), memory, and I/O (Input/Output) devices. The results are shown in Fig 5.

In Fig 5, overall, GPU has the highest resource utilization and I/O devices have the lowest. The CPU utilization of VGG16-BiLSTM-CBAM reached 78.45%, and the GPU utilization reached 85.67%, which are significantly higher than the control model. The CPU utilization of VGG16-ConvLSTM is 74.32%, and the GPU utilization is 80.54%. C3D has the lowest resource utilization, with CPU utilization of 58.34% and GPU utilization of 68.45%.

In terms of memory and I/O device utilization, VGG16-BiLSTM-CBAM has a memory utilization of 72.33% and an I/O device utilization of 65.89%. The VGG16-ConvLSTM model achieves 68.21% in memory and 60.12% in I/O devices, while C3D achieves 50.89% in memory and 48.76% in I/O devices, indicating that VGG16-BiLSTM-CBAM has higher feature storage requirements and data stream processing capabilities.

The VGG16-BiLSTM-CBAM model consumes more resources to a certain extent than other control models, but its performance improvement is sufficient to support resource investment and is suitable for the action recognition scenario in badminton in this article.

### 5.6. Ablation experiment

**Fig 6:**
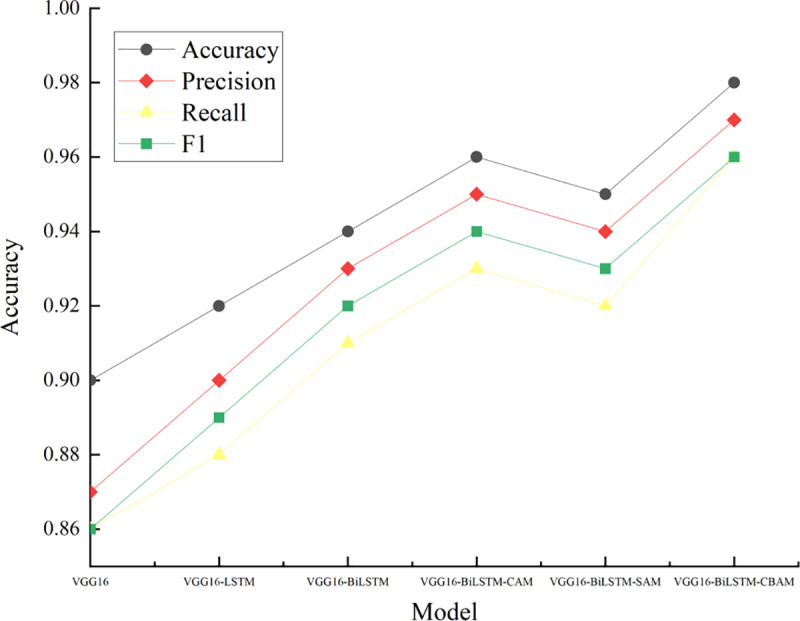
Ablation experiment results.

In this article, the experimental model is the hybrid model VGG16-BiLSTM-CBAM. Now each model can be removed in turn to explore the role of BiLSTM, CBAM and other modules in the hybrid model. The ablation experiment results are shown in Fig 6.

In Fig 6, as the model gradually removes modules, the performance gradually decreases. The accuracy of the complete model VGG16-BiLSTM-CBAM reaches 0.98, and the F1 value is 0.96. When the CBAM module is removed, the accuracy is only 0.94 and the F1 value is only 0.92. It can be seen that the CBAM module has made significant contributions to optimizing feature expression and improving classification performance. When the attention mechanism is further removed, including SAM (Spatial Attention Module), the accuracy of VGG16-BiLSTM-CAM (Visual Geometry Group 16-Bidirectional Long Short-Term Memory-Channel Attention Mechanism) is 0.96. After removing CAM (Channel Attention Mechanism), the accuracy of VGG16-BiLSTM-SAM (Visual Geometry Group 16-Bidirectional Long Short-Term Memory-Spatial Attention Module) is 0.95, indicating that channel attention is slightly better than spatial attention in improving model performance.

When the BiLSTM module is removed and only LSTM is retained, the accuracy drops to 0.92 and the F1 value is 0.89, indicating that BiLSTM is better than unidirectional LSTM in capturing bidirectional time series features. When the experiment further removes the time series feature modeling module LSTM and only retains VGG16, the accuracy drops to 0.9 and the F1 value is only 0.86.

In summary, the BiLSTM module is the core of time series modeling, while the attention mechanism plays a key role in optimizing feature distribution and weight allocation. The combination of the two significantly enhances the classification performance of the model.

### 5.7. Verification of generalization ability in daily exercise scenarios of teenagers

This paper collects data from the actual daily exercise scenarios of multiple young badminton players, constructs a new badminton action recognition dataset, and verifies it on the VGG16-BiLSTM-CBAM model. The verification indicators include accuracy, precision, recall, and F1 value. The generalization ability verification results in daily exercise scenarios of teenagers are shown in Table 3. The test environment includes:

**Table 3:** Generalization ability verification results in daily exercise scenarios of teenagers.

Daily exercise scene	Accuracy	Precision	Recall	F1
Indoor stadium	0.98	0.97	0.96	0.96
Home backyard	0.95	0.94	0.93	0.93
School outdoor stadium	0.96	0.96	0.94	0.95
Community sports field	0.93	0.92	0.89	0.90

Indoor stadium: standard sports facilities, relatively stable lighting and noise environment.

Home backyard: non-standard venue, more lighting and background interference.

School outdoor stadium: open venue, with strong lighting and external noise interference.

Community playground: more crowded and noisy environment, large changes in light and background.

In Table 3, the VGG16-BiLSTM-CBAM model has strong generalization ability in the daily exercise scene of teenagers and can perform stably in various environments. In the standard indoor stadium, the model’s accuracy and F1 value both reached the best, 0.98 and 0.96 respectively. As a non-standard venue, the family backyard has more light and background interference, which leads to a decrease in recognition performance, but the accuracy remains at 0.95, and other indicators also perform well. The open venue and external interference of the school outdoor stadium are slightly larger, but the model can still maintain a high recognition performance, with an accuracy of 0.96 and an F1 value of 0.95. Due to the large changes in light and background in the community sports field, the recognition performance has declined, with the accuracy dropping to 0.93 and the F1 value dropping to 0.90. The performance is relatively weak, but it can still maintain a high accuracy and recognition ability. Overall, the performance of the VGG16-BiLSTM-CBAM model in different daily exercise scenes proves its good generalization ability, can adapt to a variety of complex actual environments, and provides reliable technical support for youth sports recognition.

### 5.8. Statistical significance test

To verify whether the performance improvement of the VGG16-BiLSTM-CBAM model is statistically significant between different control models, this paper will conduct a T test analysis to compare the differences in accuracy, precision, recall and F1 values between VGG16-BiLSTM-CBAM and other control models VGG16-ConvLSTM, ResNet, InceptionV3 and C3D, and conduct a significance test to ensure the robustness of the model performance improvement. The results of the statistical significance test are shown in Table 4.

**Table 4:** Results of statistical significance test.

Model comparison	Accuracy T value	Precision T value	Recall T value	F1 value T value P value	Model comparison
VGG16-BiLSTM-CBAM vs VGG16-ConvLSTM	2.14	3.21	2.76	2.55	0.02
VGG16-BiLSTM-CBAM vs ResNet	4.25	4.11	3.87	4.02	0.01
VGG16-BiLSTM-CBAM vs InceptionV3	3.99	3.89	3.72	3.85	0.03
VGG16-BiLSTM-CBAM vs C3D	5.1	4.85	4.92	5.05	0.01

Experimental design:

Null hypothesis (H0): There is no statistically significant performance difference between VGG16-BiLSTM-CBAM and the control model.

Alternative hypothesis (H1): VGG16-BiLSTM-CBAM is significantly better than the control model in performance.

The experiment uses T test for pairwise comparison to test the differences in accuracy, precision, recall and F1 values between VGG16-BiLSTM-CBAM and other models. The significance level was set at α = 0.05.

In Table 4, it can be seen that among all the control models, the differences in accuracy, precision, recall and F1 value of VGG16-BiLSTM-CBAM are statistically significant, and the P values are all less than 0.05. This shows that the performance improvement of the VGG16-BiLSTM-CBAM model in badminton motion recognition is significant and can effectively surpass other traditional models. The performance improvement of the VGG16-BiLSTM-CBAM model is not only superior in practice, but also statistically robust, and can be reliably used for youth badminton motion recognition.

## 6. Experimental discussion

This study analyzes the performance of VGG16-BiLSTM-CBAM and a variety of control models. The results show that VGG16-BiLSTM-CBAM has significant advantages in badminton motion recognition. Its accuracy is as high as 0.98, significantly better than VGG16-ConvLSTM, ResNet, InceptionV3 and other control models. VGG16-BiLSTM-CBAM showed strong robustness in different noise and light intensity environments, with the highest accuracy of 0.97 in a medium light environment of 500 lux. Ablation experiments further revealed the key role of BiLSTM and CBAM modules in model performance, with BiLSTM outperforming unidirectional LSTM in capturing bidirectional temporal features, and the CBAM attention mechanism significantly improving the model’s classification ability by strengthening channel and spatial features. The training time and parameter count of VGG16-BiLSTM-CBAM are slightly higher, but its response time is only 45ms, and its resource utilization is relatively high, which can achieve real-time and efficient action recognition in actual scenarios.

The results of this study are of great significance for the automatic analysis of badminton sports behavior among young people. The VGG16-BiLSTM-CBAM model improves the accuracy and stability of action recognition by integrating deep feature extraction, bidirectional temporal modeling and attention mechanism, providing a feasible technical solution for real-time monitoring and evaluation of sports actions. The research can help athletes optimize their movements and reduce potential sports risks by reducing recognition errors. The robustness test of this study in complex environments provides a reliable basis for the deployment of the model in actual sports scenarios.

In this paper, in addition to posture correction and safety monitoring, the experimental model has great potential for application in sports training evaluation and rehabilitation therapy. The VGG16-BiLSTM-CBAM model can provide real-time feedback for sports training by accurately identifying the movement characteristics of athletes, helping coaches evaluate the technical level of athletes and optimize training programs. In rehabilitation therapy, the model can be used to monitor the patient’s recovery progress, especially in the process of motor function recovery, to help doctors evaluate the patient’s movement quality and movement standardization in real time, and better guide rehabilitation training.

The VGG16-BiLSTM-CBAM model used in this paper provides an effective solution for badminton action recognition and lays a technical foundation for the future development of intelligent youth sports auxiliary tools. The study verified the synergy between temporal modeling and attention mechanism in DL, and provided new ideas for further optimizing motion recognition technology. Future work can focus on lightweight design of the model to meet the application needs of resource-constrained scenarios and expand its applicability in the recognition of various motion behaviors.

There are some shortcomings in this paper:

(1) The VGG16-BiLSTM-CBAM model requires high computing resources during training and inference, especially in terms of the number of parameters and training time, which makes it difficult to deploy the model on resource-constrained devices. Future work will focus on the lightweight design of the model to reduce unnecessary computing and memory consumption. Correspondingly, techniques such as knowledge distillation, pruning or quantization can be used to reduce the size and computational complexity of the model, making it more efficient in embedded devices or real-time applications.
(2) The VGG16-BiLSTM-CBAM model performs well in the inference stage with a response time of 45ms, but it still needs further optimization for some real-time motion monitoring systems or motion analysis in high-dynamic environments. In order to meet more stringent real-time requirements, more efficient inference architectures or optimization algorithms will be explored in the future, and more efficient CNN architectures or lightweight LSTM variants will be used to further reduce latency and maintain high recognition accuracy.
(3) This study was only verified in the specific sport of badminton, and the dataset used was not specifically for youth sports. Future research will be extended to other sports, such as basketball, table tennis, etc., and the model will be applied to the recognition of motion behaviors of different groups of people to verify the effectiveness and robustness of the model in multiple sports and populations.

## 7. Conclusions

This paper adopts a DL method based on VGG16, BiLSTM and CBAM modules to improve the accuracy of youth badminton motion recognition. The study optimizes the VGG16 model, embeds the CBAM module to enhance feature representation, and combines BiLSTM to capture temporal dependency information. The experimental results show that the VGG16-BiLSTM-CBAM model can effectively improve the performance of motion recognition in youth sports activities and provide technical support for the correction and safety monitoring of sports postures. This study has achieved some achievements, but there are also certain limitations. The model has a large number of parameters, which leads to a long training time, and only badminton is studied in daily sports activities. Future research can promote the application of DL in youth sports activities by introducing more types of sports data, improving the diversity and robustness of the model, and optimizing the model.

## Supporting information

S1 Data (XLSX)
